# Tumorigenic mesenchymal clusters are less sensitive to moderate osmotic stresses due to low amounts of junctional E-cadherin

**DOI:** 10.1038/s41598-021-95740-x

**Published:** 2021-08-11

**Authors:** Danahe Mohammed, Chan Young Park, Jeffrey J. Fredberg, David A. Weitz

**Affiliations:** 1 John A. Paulson School of Engineering and Applied Sciences, Harvard University, Cambridge, MA USA; 2 Department of Physics, Harvard University, Cambridge, MA USA; 3 Harvard T.H. Chan School of Public Health, Boston, MA 02115 USA

**Keywords:** Biophysics, Motility

## Abstract

The migration of tumorigenic cells is a critical step for metastatic breast cancer progression. Although the role of the extracellular matrix in breast cancer cell migration has been extensively described, the effect of osmotic stress on the migration of tumor breast cohorts remains unclear. Most of our understanding on the effect of osmotic stresses on cell migration comes from studies at the level of the single cell in isolation and does not take cell–cell interactions into account. Here, we study the impact of moderate osmotic stress on the migration of cell clusters composed of either non-tumorigenic or tumorigenic cells. We observe a decrease in migration distance and speed for non-tumorigenic cells but not for tumorigenic ones. To explain these differences, we investigate how osmotic stress impacts the mechanical properties of cell clusters and affects their volumes. Our findings show that tumorigenic mesenchymal cells are less sensitive to osmotic stress than non-tumorigenic cells and suggest that this difference is associated with a lower expression of E-cadherin. Using EGTA treatments, we confirm that the establishment of cell–cell adhesive interactions is a key component of the behavior of cell clusters in response to osmotic stress. This study provides evidence on the low sensitivity of mesenchymal tumorigenic clusters to moderate osmotic stress and highlights the importance of cadherin-based junctions in the response to osmotic stress.

## Introduction

A fundamental challenge is to understand how cell mechanical properties are impacted by the microenvironment. Recent research shows that mechanical properties of cells and the tumor microenvironment can influence cell behavior, tumor growth and dynamics, in ways that remain poorly understood^1–7^. Cell behavior can be influenced by the matrix rigidity^8–10^, the confinement^11–14^ or the topography^15–17^. In the case of cancer, the properties of the tumor matrix are also impacted by the environment which determines how cancer cells polarize, adhere, contract and migrate, thereby regulating their invasiveness^18^. One of the important parameters of the microenvironment for cell regulation is osmotic pressure^19–21^. Many studies have shown that compressive forces induced by osmotic pressure can critically impact cancer cell migration and proliferation, and their growth in the form of tumor spheroids^1,5,22,23^. In addition, Stroka and coworkers reported that osmotic gradients in the extracellular space influence cell migration by regulating ion/water transport proteins. Assuming that protein transport could be affected by changes in extracellular osmolality, other studies suggest that osmotic pressure could be a driving force for cell migration during cancer progression^23,24^. Moreover, it was shown that extracellular osmolarity or modification of specific ion concentrations can modulate the mechanical properties of the cell through volume changes, that in turn may impact important cellular processes, such as mitosis, mitochondrial functions or DNA repair^25–27^. In this context, recent works suggest that gap junctions can be a key component of a mechano-osmotic model that can describe how cell volume is regulated within tumoral clusters in response to osmotic pressure^28^.

The osmotic pressure is determined by the concentration of ions and proteins within the cell microenvironment. Controlling osmotic pressure has various impacts on cell behavior, such as modification of the volume and morphology as well as changes in cell motility^23,29–32^. Moreover, major changes in the functioning of cells are observed^14,33–35^. Hyperosmotic stress alters a variety of cellular processes, for example, it disrupts the cytoskeleton structure^14,36,37^, induces chromatin remodeling^38,39^, and triggers cell cycle arrest^40,41^ and apoptosis^42,43^. In a pathological context, recent studies have shown that serum albumin, whose main function is to regulate the osmotic pressure of blood, is associated with better survival during cancer^14,14,44^. The transmigration capability of cancer cells, which enables cancer cells to invade surrounding tissue, decreases when a low osmotic pressure is applied (~ 1 kPa)^1^. In addition, osmotic pressure has an impact on stem cell fate, suggesting that changes in cell volume due to osmotic pressure alter physiological parameters like stem-cell differentiation^35^. Taken together, these observations have led to the recognition of osmotic pressure as an important parameter in physiological and pathological situations and demonstrate a clear link among cell behavior, cancer and osmotic pressure. However, most previous work used levels of osmotic stress^33,34,45–47^ far from the physiological and pathological ranges^14,48–51^. Thus, the mechanism by which osmotic stress modulates cell migration and the precise impact it has on tumorigenic cells remains poorly understood. Further, most previous studies of the role of osmotic stress were performed at the single-cell level^1,35^ and do not take into account the modulation of cell–cell adhesions among tumor cells. Cell–cell interactions are crucial for tissue integrity, collective cell migration and cancer propagation.

In this paper, we determine the effect of moderate osmotic stresses on the migration of minimal cell clusters and investigate the role of cell–cell junctions. To apply hyperosmotic stress, we add 300-Da polyethylene glycol (PEG 300) directly to the medium. We demonstrate that when non-tumorigenic epithelial cells are cultured under osmotic stress, not only the cell volume but also cell motility decrease. By contrast, this behavior is not observed with tumorigenic mesenchymal cells, which are shown to be less affected by osmotic pressure. By inhibiting cell–cell adhesions in non-tumorigenic cells we show that they become less affected by osmotic stresses, similar to what is observed in tumorigenic cells which have lower cell–cell adhesions. These observations reveal a surprising and previously unidentified role of cell–cell adhesions in the regulation of cell volume and migration in response to variations of osmotic pressure.

## Results

### Osmotic stress modulates migration distance and speed of non-tumorigenic epithelial cells but not tumorigenic mesenchymal breast cells

**Figure 1:**
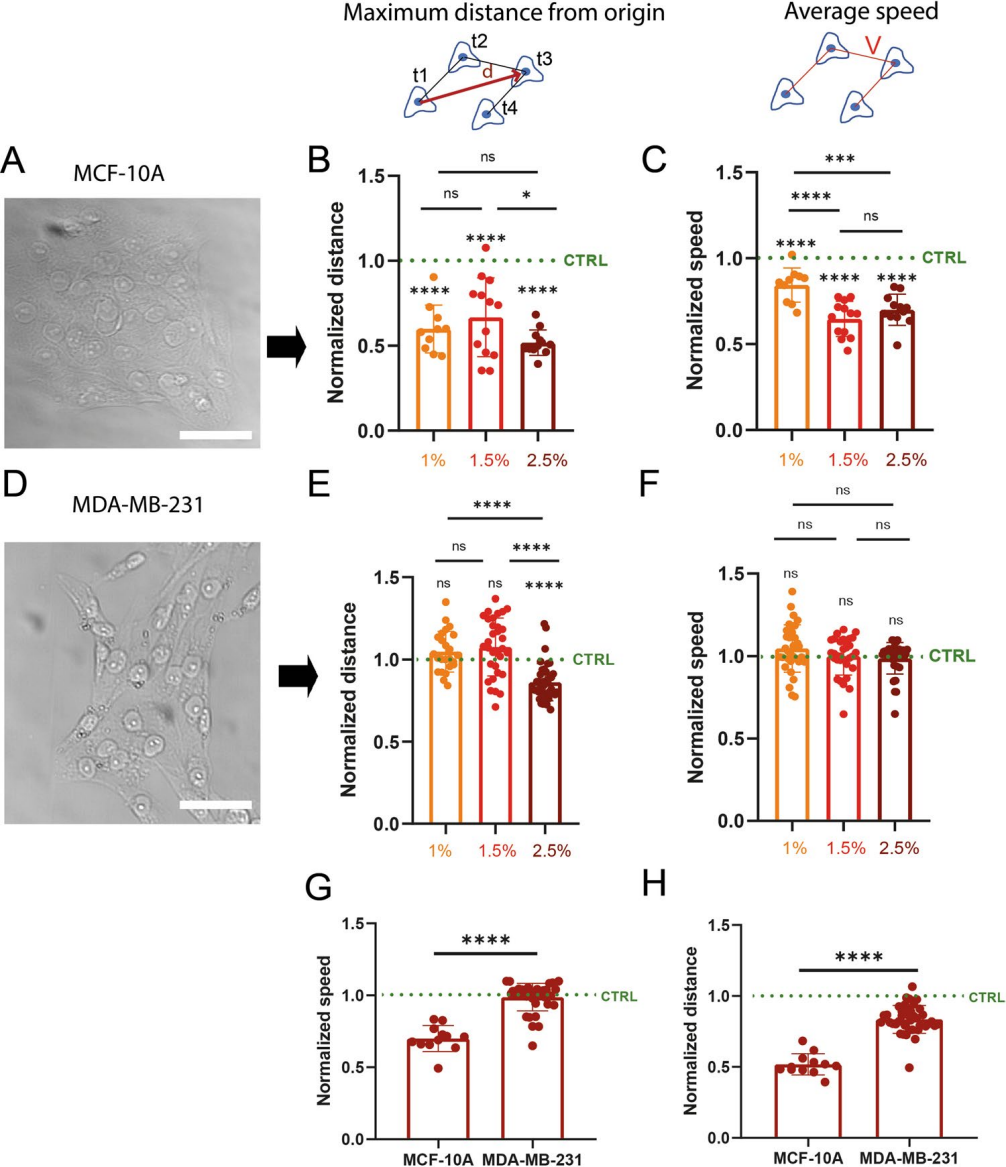
Osmotic-stress modulates migration and speed of non-tumorigenic epithelial cells, but not those of tumorigenic breast mesenchymal cells. Brightfield images of (**A**) MCF-10A and (**D**) MDA-MB-231 cell clusters. Scale bars correspond to 50 µm. Normalized maximum migration distances of (**B**) MCF-10A and (**E**) MDA-MB-231 treated with 1% (yellow), 1.5% (red) and 2.5% (dark red) PEG concentrations. For each cell type and PEG concentration, maximal distances of migration are normalized by maximum distance of migration of control cells. Normalized migration speeds of (**C**) MCF10A and (**F**) MDA-MB-231 treated with 1% (yellow), 1.5% (red) and 2.5% (dark red) PEG concentrations. For each cell type and PEG concentration, migration speeds are normalized by migration speeds of control cells. (**G**) Normalized migration speeds and (**H**) normalized migration distances for MCF-10A and MDA-MB-231 treated with 2.5% PEG. 10 ≤ n ≤ 13 independent experiments for MCF10A and 24 ≤ n ≤ 40 for MDA-MB-231. *0.01  < *p*< 0.05, **0.001 < *p* < 0.01, ***0.0001 < *p* < 0.001, ****p < 0.0001 and n.s. non-significant.

To investigate whether osmotic pressure can affect the migration of breast cells, we used two different cell lines: a non-tumorigenic epithelial breast line (MCF-10A, Fig. 1A–C) and a tumorigenic mesenchymal breast line (MDA-MB-231, Fig. 1D–F). Both cell lines were cultivated on glass substrates coated with fibronectin (FN). Fibronectin was selected as a model extracellular matrix (ECM) protein because all cell types used in our study show common integrins, such as α5β1 that bind to fibronectin. After 8 h in culture with fresh media, we add three different concentrations of PEG 300 (Supplementary Fig. S1A–B) into the culture medium to exert well-controlled moderate osmotic stresses on cell cultures ranging from ~ 128 to ~ 231 kPa, as observed in physiological situations^48–51^. The motility of the different cell lines in response to osmotic stresses is then observed with time-lapse microscopy. For each cell line, experiments are started with an initial acquisition of 8 h under isotonic conditions (culture medium), representing the control. Then, a PEG solution of 1%, 1.5% or 2.5% (v/v) is added to the culture medium and an additional time-lapse acquisition of 8 h is collected (Supplementary Movies S1–S6). We estimated the viability of cells treated with PEG and do not observe that moderate osmotic pressures impact cell viability (Supplementary Fig. S2). To avoid any artefacts and to ensure the lowest variability in our analysis, the migration distance and speed determined for each PEG concentration are normalized by the values obtained from the control experiments performed on the same culture. This normalization enables us to determine the variation of migration distance and speed from the control for each PEG concentration. Interestingly, we find that the maximum distance from the origin (Fig. 1B) and the average migration speed (Fig. 1C) of MCF-10A cells decreases significantly in response to moderate osmotic stress. The normalized distance and average speed decrease monotonically with increasing PEG concentration, reaching values of 9.84 ± 1.44 µm (n = 13) and 0.192 ± 0.026 µm/min (n = 7) respectively at a concentration of 2.5% (v/v). We observe a slight effect of PEG concentration between PEG 1% and PEG 1.5–2.5% but we don’t observe an effect of PEG concentration on maximum migrating distance suggesting that MCF-10A are sensitive to very low osmotic stress (130 kPa). Surprisingly, our findings demonstrate that the migration parameters of MDA-MB-231 cells are not affected by osmotic stresses for PEG concentrations ranging from 1% to 2.5% (v/v) (Fig. 1E, F). Interestingly, the differences in migratory speed (Fig. 1G) and distance (Fig. 1H) are statistically different at 2.5% (v/v) PEG for the two cell lines. These results demonstrate that tumorigenic mesenchymal breast cells are more resistant to osmotic stress changes than non-tumorigenic breast epithelial cells.

### The rigidity of breast cell lines is not affected by osmotic stresses

**Figure 2:**
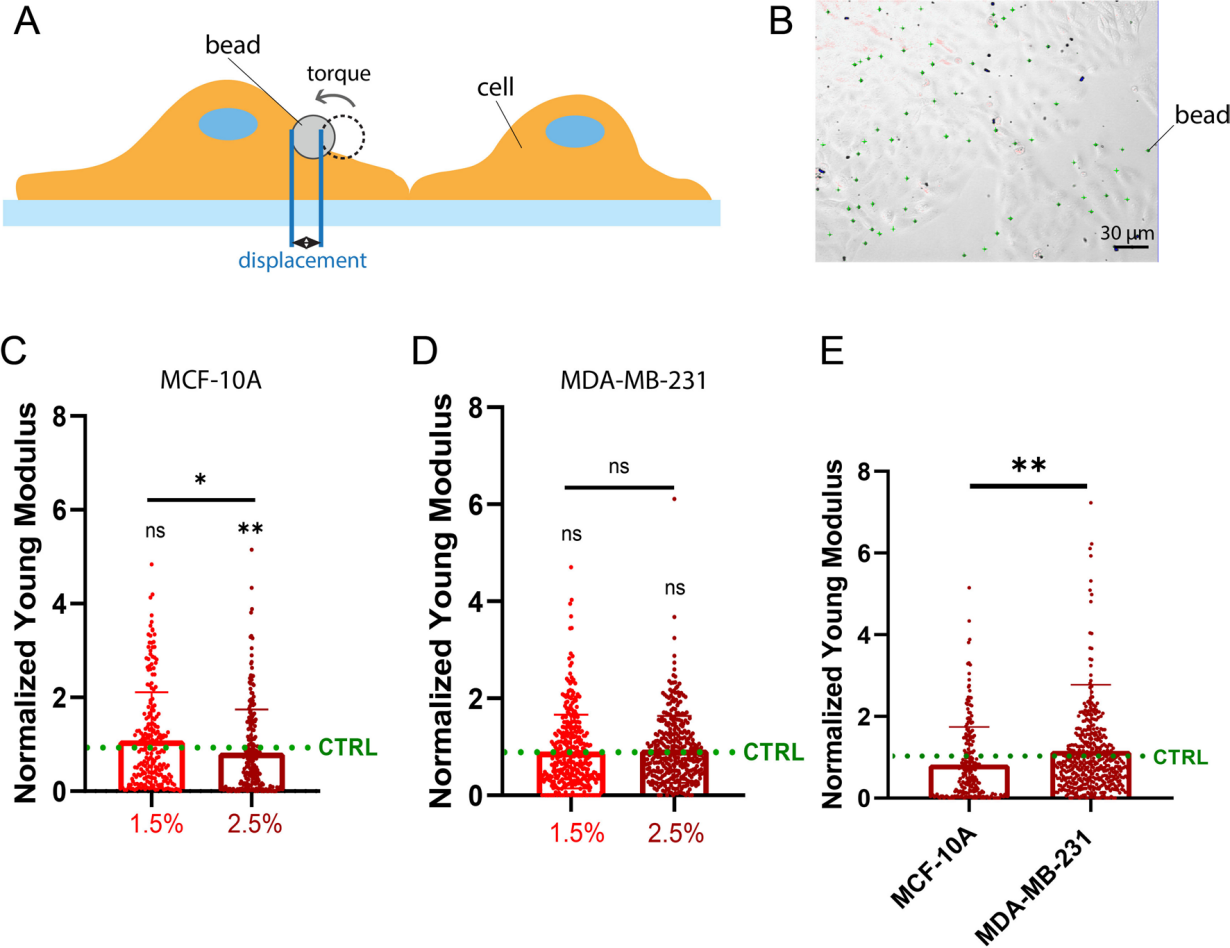
The rigidity of breast cell lines is not affected by osmotic stress. (**A**) Schematic representation of OMTC measurements. (**B**) Typical DIC images of OMTC experiment. Elastic modulus with different concentrations of PEG 1.5% (red) and 2.5% (dark red) normalized by those under isotonic condition for (**C**) MCF-10A and (**D**) MDA-MB-231 cells. (**E**) Comparison of normalized elastic modulus for the two cell lines exposed to 2.5% PEG with 70 ≤ n ≤ 140 for all experiments. *0.01 < *p* < 0.05, **0.001 < *p* < 0.01, ****p < 0.0001 and n.s. non-significant.

To help understand the differences in migration among non-tumorigenic (MCF10A) and tumorigenic breast cells (MDA-MB-231) in response to moderate osmotic stress, we measure the change in cell stiffness of the two cell lines in response to osmotic stress at 1.5% and 2.5% (v/v) using optical magnetic twisting cytometry (OMTC)^52^ (Fig. 2A,B). Indeed, the cellular stiffness or rigidity, which is usually expressed as an elastic modulus in Pascals, is an important cellular characteristic that characterize the tridimensional (3D) reorganization of the cell cytoskeleton in response to physico-chemical changes of the cell microenvironment. Previous works have shown that the cell stiffness matches the substrate rigidity^53,54^, cell stiffness is involved in stem cell behavior^55^ and a low cell stiffness promotes cell migration^56^. Under osmotic compression, water efflux can increase leading to the buckling of cytoskeletal filaments, thereby reducing the cell stiffness and consequently, this cell rigidification due to water efflux with osmotic pressure can have an impact on cell migration^35,52,57,58^. In addition, some of us have shown that cell stiffness can increase with osmotic pressure in different cell types, based on cell volume changes through water efflux^35^ and can induce glass transition because compression dramatically slows intracellular relaxation processes^52^. Based on these findings, we examined whether the cellular stiffness of normal and tumorigenic tissue can change with the osmotic pressure with regard to their migratory behavior. Similar to the migration parameters, we normalize the cell stiffness in response to 1.5% and 2.5% (v/v) PEG with the baseline stiffness measured on the same cells in the absence of PEG. We observe that the individual stiffness of MCF-10A (Fig. 2C) and MDA-MB-231 (Fig. 2D) cells were not affected by moderate osmotic stresses at 1.5% or 2.5% of PEG. Interestingly, MCF-10A were statistically softer that MDA-MB-231 at 2.5% of PEG (Fig. 2E). Taken together, our results suggest that the cell rigidity is not sufficiently affected by osmotic pressure to be considered as a key component of the decrease in migration speed under moderate osmotic pressures.

### Moderate osmotic stress leads to a reduction in cell volume, but tumorigenic breast mesenchymal cells are less sensitive to osmotic stress

**Figure 3:**
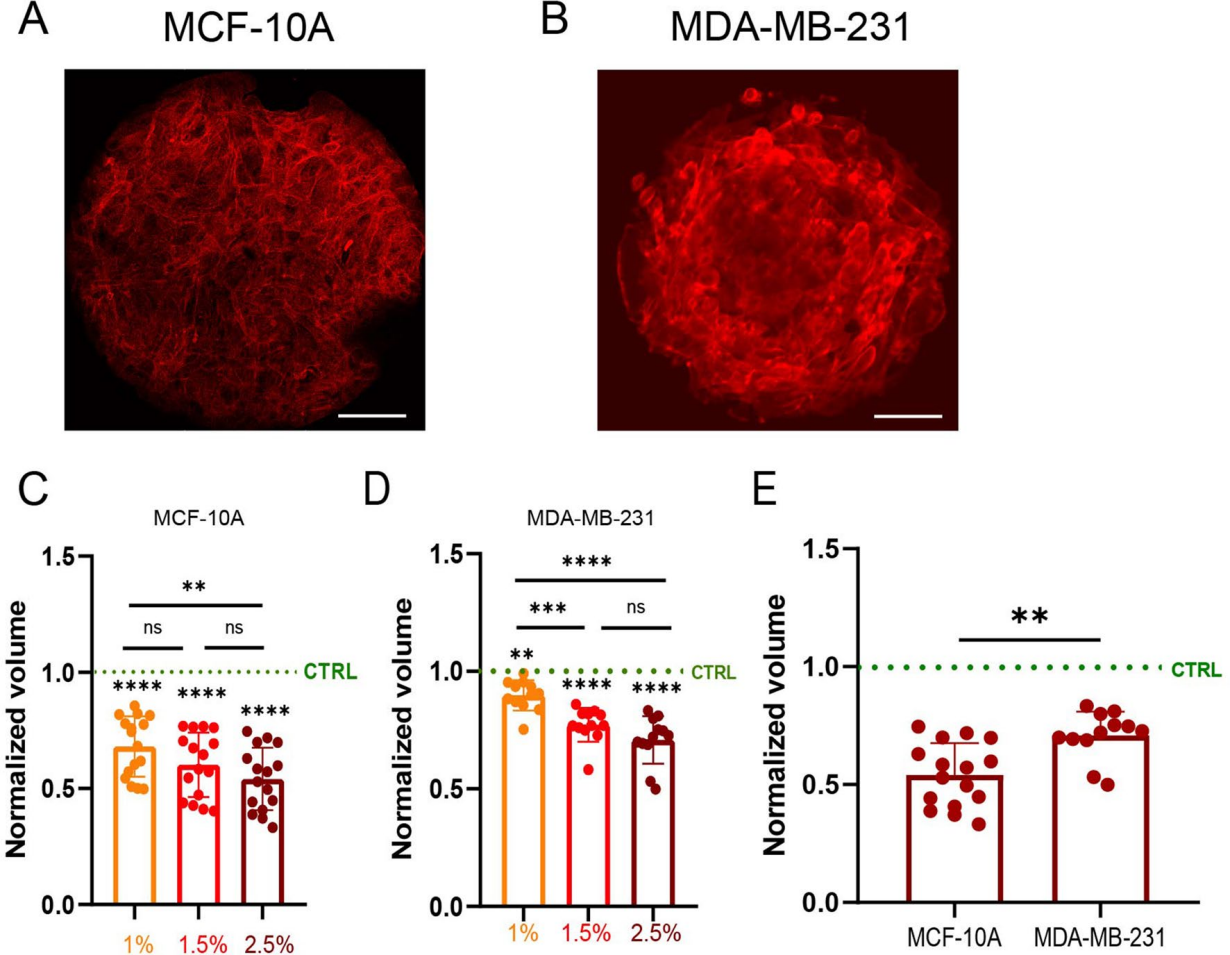
Moderate osmotic stress leads to cell volume loss, but tumorigenic breast mesenchymal epithelial cells are less sensitive to osmotic stress. Immunofluorescence images of (**A**) MCF-10A and (**B**) MDA-MB-231 minimal tissues grown on circular FN micropatterns of 200 µm in diameter. Actin is stained in red. Scale bar corresponds to 40 µm. Normalized volumes of (**D**) MCF-10A and (**E**) MDA-MB-231 micropatterned tissues treated with different PEG concentrations: 1% (yellow), 1.5% (red) and 2.5% (dark red). For each cell type, the volumes of PEG-treated tissues are normalized with the volume of micropatterned tissues. n = 16 (MCF-10A), n = 12 (MDA-MB-231). *0.01 < *p* < 0.05, **0.001 < *p* < 0.01, ***0.0001 < *p* < 0.001, ****p < 0.0001 and ns non-significant.

When isolated cells are subjected to an external osmotic pressure, their volume decreases due to water efflux, and their stiffness increases^35^. The internal osmotic pressure of a cell is regulated by the concentration of ions and small proteins and must adapt to changes in the external osmotic pressure to eliminate any gradient in pressure across the cell membrane. This can result in a significant change in subcellular macromolecular density. To further investigate whether moderate osmotic stresses applied to cell monolayers can lead to cellular volume changes, we measure by confocal microscopy the volume of minimal tissues composed of either MCF-10A or MDA-MB-231 cells. To standardize our measurements, we form tissues of MCF-10A (Fig. 3A) and MDA-MB-231 (Fig. 3B) by plating cells on circular micropatterns of FN. Micropatterns enable control of the size of the minimal tissues and their cellular density, thus limiting the variability between clusters. By labeling the cytoplasm in live conditions with cell tracker, we determine the cell volume of the whole circular tissues under isotonic conditions (culture medium) and then in response to moderate osmotic stresses of 1%, 1.5% and 2.5% (v/v) of PEG. We normalize the volumes of the cells subjected to PEG by those of the control for each cell line to highlight variations of cell volume. Osmotic stress leads to significant decreases in volume for MCF-10A (~ 46%, Fig. 3C), whereas only a moderate decrease in volume is observed for tumorigenic breast cells, MDA-MB-231 (~ 29%, Fig. 3D). We found therefore that MDA-MB-231 cells are less sensitive to moderate osmotic stress than MCF-10A cells (Fig. 3E), suggesting that tumorigenic mesenchymal cells are more robust to variations of osmotic pressure than non-tumorigenic epithelial cells.

### The establishment of cell–cell interactions leads to a larger volume loss in response to osmotic stresses

**Figure 4:**
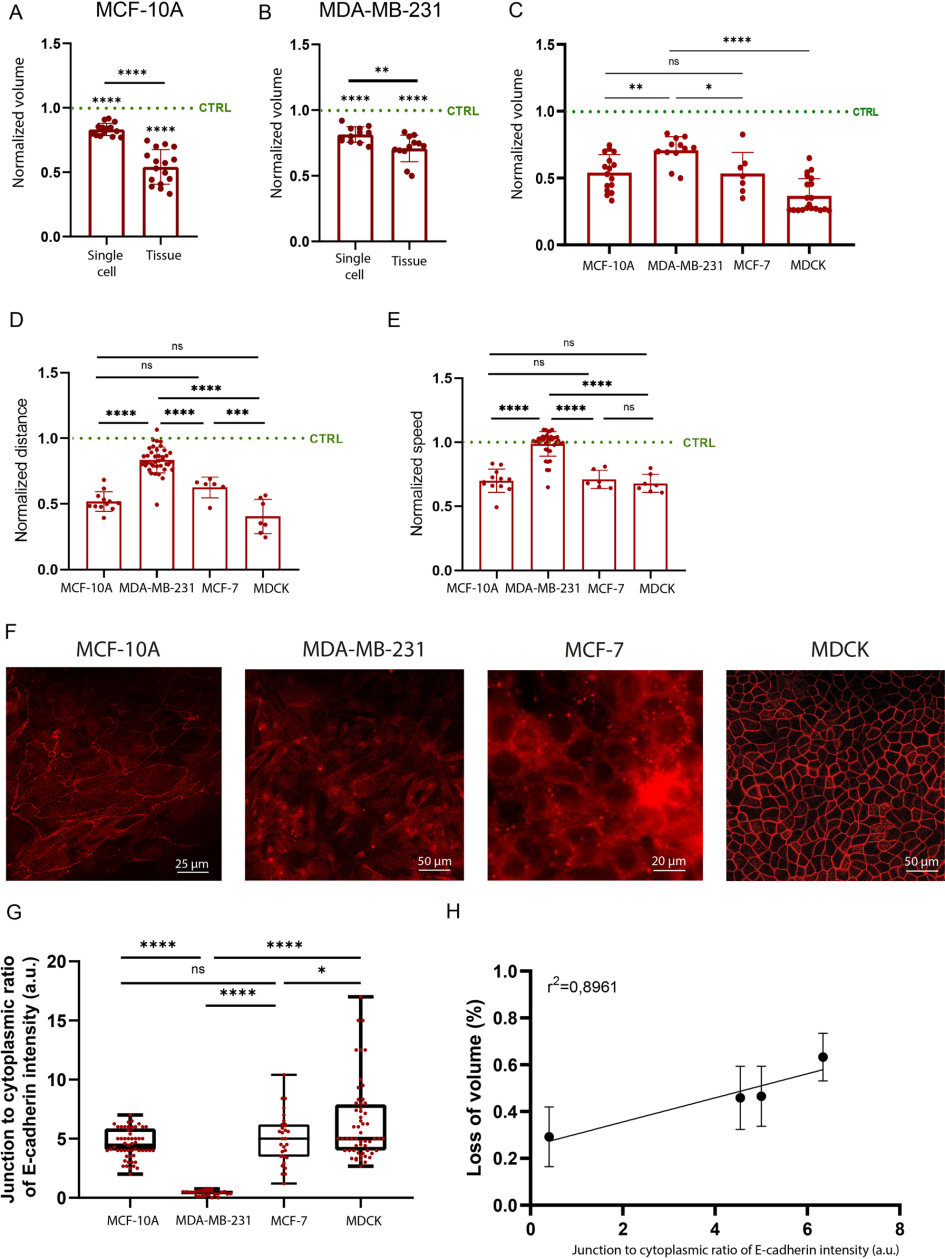
The establishment of cell–cell interactions modulates the volume loss in response to osmotic stress. Comparison of the cell volume of single cells and micropatterned tissues for (**A**) MCF-10A and (**B**) MDA-MB-231 treated with PEG 2.5%. For each cell type, the volumes of PEG-treated tissues are normalized by the volume of micropatterned tissues in isotonic condition. (**C**) Comparison of Normalized volume treated with PEG 2.5% for MCF-10A, MDA-MB-231, MCF-7 and MDCK. For each cell type, the volumes of PEG-treated tissues are normalized by the volume of micropatterned tissues in isotonic condition. (**D**) Normalized maximum distance and (**E**) normalized speed of MCF-10A, MDA-MB-231, MCF-7 and MDCK cells. For each cell type, maximal distances of migration or speed are normalized by maximum distance of migration or speed of control cells. (**F**) Staining of E-cadherin for of MCF-10A, MDA-MB-231, MCF-7 and MDCK cells. (**G**) Quantification of the junction to cytoplasm ratio of E-cadherin intensity for of MCF-10A, MDA-MB-231, MCF-7 and MDCK. (**H**) Linear relation (R^2^ = 0.8961) between the cell volume loss (%) and the junction to cytoplasm ratio of E-cadherin intensity. Cells exhibiting higher cadherin fluorescence intensity show larger volume loss. n = 15 (MCF-10A single cells), n = 16 (MCF-10A tissues), n = 12 (MDA-MB-231 cells), n = 12 (MDA-MB-231 tissues). *0.01 < *p* < 0.05, **0.001 < *p* < 0.01, ***0.0001 < *p* < 0.001, ****p < 0.0001 and ns non-significant.

We investigate the role of cell–cell adhesive interactions to understand the mechanism leading to large volume variations loss in clusters of MCF-10A and MDA-MB-231 cells. Indeed, we compare the volume of individual MCF-10A and MDA-MB-231 cells with MCF-10A and MDA-MB-231 tissues in response to moderate osmotic stress. Our results show a PEG induced volume loss for single cells, which is statistically similar for MCF-10A (~ 17%, Fig. 4A) and MDA-MB-231 (~ 19%, Fig. 4B) cells. Interestingly, there is a smaller difference in cell volume loss between single cells and cell clusters for tumorigenic cells (Fig. 4B) than for non-tumorigenic epithelial cells (Fig. 4A), suggesting that cell–cell adhesions may be a key player of the volume modulation in response to osmotic stress. To investigate this hypothesis, we use two others cell lines: the MCF-7 tumoral breast cell line^59–61^ and MDCK cells, which are commonly used as strong cell–cell adhesion model^62,63^. Indeed, the MCF-10A cell line is derived from benign proliferative breast tissues, and MCF-10A cells are not tumorigenic and do not express estrogen receptors^64^. MDA-MB-231 is an human breast cancer cell line obtained from a metastatic mammary adenocarcinoma^65–67^. MCF-10A express good level of cell–cell adhesion protein such as E-cadherin but fail to form tight junctions^64,68–71^. Interestingly, MDA-MB-231 cells have severely poor cell–cell adhesion and a lack of E-cadherin^73^. MCF-7 is also a tumorigenic breast cell line which expresses E-cadherin^59–61^. MDCK is a perfect model for stronger cell–cell adhesion with a full display of E-cadherin junctions and tight junctions which are lacking in MCF-10A^62,63^.

Using these two other cell lines, our results show that osmotic stress leads to large volume decrease for all the cell lines expressing E-cadherin: non-malignant MCF-10A, malignant MCF-7 and MDCK cells which are characterized by a stronger cell–cell interactions (Fig. 4C, Supplementary Fig. S3). However, we observed a lower volume loss for the MDA-MB-231 tumorigenic breast mesenchymal cells, characterized by a low expression of E-cadherin (Fig. 4C).

To confirm our findings, we quantify the maximum normalized distance (Fig. 4D) and speed (Fig. 4E) of MCF-7 and MDCK cells treated with PEG 2.5% to compare their migrating behavior with MCF-10A (Fig. 1B,C) and MDA-MB-231 (Fig. 1E,F). Our results show that the migratory behavior of cell types establishing E-cadherin cell–cell interactions (MCF-10A, MCF-7 and MDCK) is sensitive to moderate osmotic pressure, whereas MDA-MB-231 cells characterized by a low E-cadherin interactions is less sensitive to osmotic pressure. To further understand these results, we determine the contrast of E-cadherin between cell–cell junctions and the cytoplasm by immunostaining experiments (Fig. 4F), as determined in previous studies^74^. As shown in Fig. 4G, MDCK tissues exhibit the largest E-cadherin intensity at cell–cell junctions, whereas MCF-10A and MCF-7 express a similar level of E-cadherins and MDA-MB-231 show no detectable fluorescence signal at cell–cell junction. Furthermore, we demonstrate that the volume loss in PEG-treated tissues is linearly correlated with the intensity of E-cadherin (Fig. 4H, R^2^ = 0.8961), confirming that the low amount of cadherin-based junctions in mesenchymal tissues (MDA-MB-231) results in a larger resistance to volume loss.

### The effect of osmotic stresses on the migration velocity of epithelial cells is lowered by disrupting cell–cell adhesions

**Figure 5:**
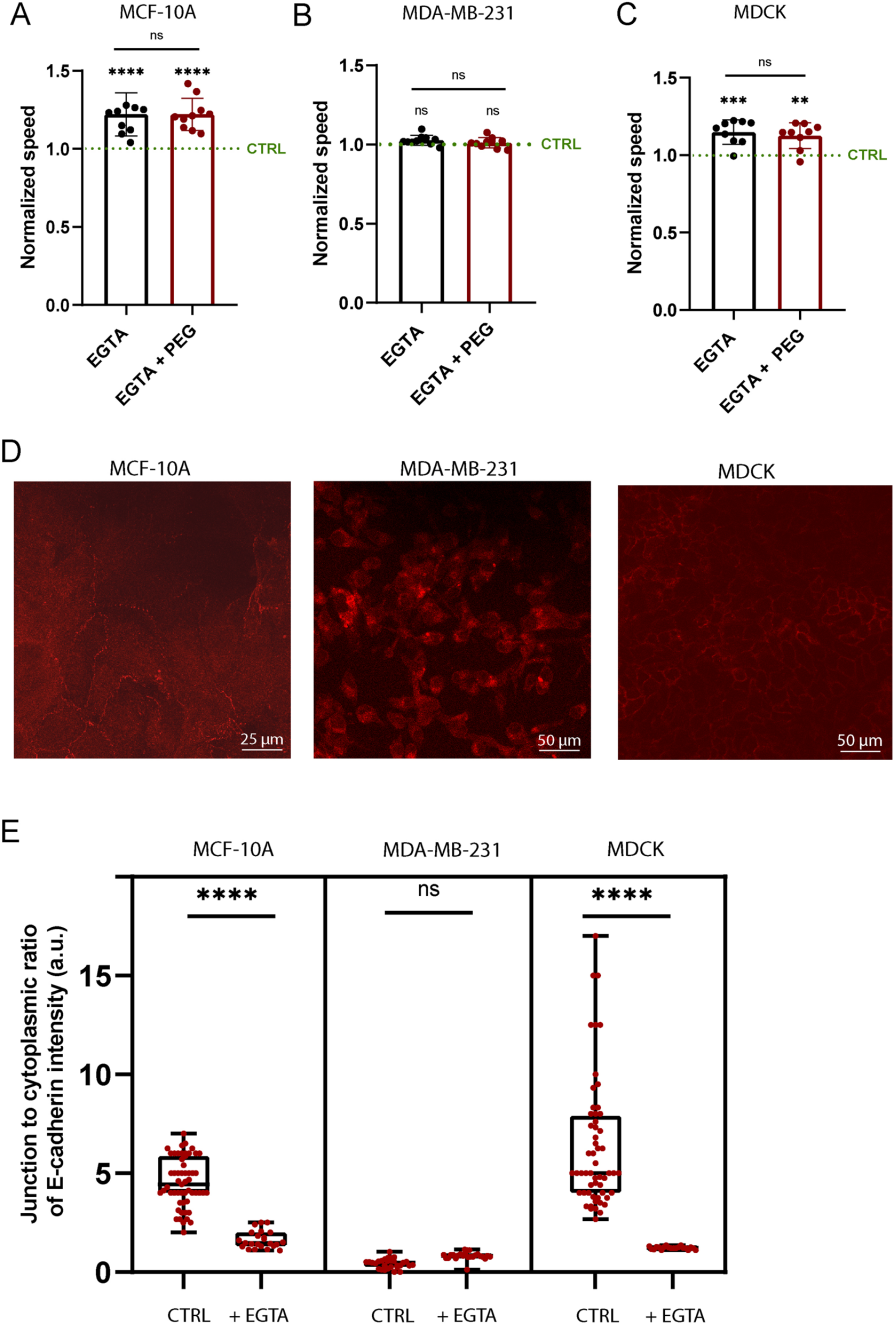
The effect of osmotic stress on the migratory behavior of epithelial cells is reduced by disrupting cell–cell adhesions. Average cell speed after EGTA treatment and after EGTA treatment followed by PEG 2.5% osmotic stress, both normalized with control values (without PEG and EGTA) for (**A**) MDCK, (**B**) MCF-10A and (**C**) MDA-MB-231 cell clusters respectively, with n = 9 (MDCK), n = 13 (MCF10A) and n = 10 (MDA-MB-231). (**D**) Immunostaining of E-cadherin after EGTA treatment for MCF10A, MDA-MB-231 and MDCK. (**E**) Quantification of the junction to cytoplasm ratio of E-cadherin intensity before and after EGTA treatment for MCF10-A, MDA-MB-231 and MDCK. *0.01 < *p* < 0.05, **0.001 < *p*  < 0.01, ***0.0001 < *p* < 0.001, ****p < 0.0001 and n.s. non-significant.

To further confirm the role of the cell–cell adhesive interactions on the sensitivity of epithelial tissues to moderate osmotic stress, we perform time-lapse experiments on EGTA-treated tissues which disrupts cell–cell junctions^75,76^ and thus converts the cell tissue into a group of isolated cells (Supplementary Movies S7–S8). We acquire 8 h of images under isotonic conditions in culture medium to obtain a control, and then add EGTA solution at 5 mM and acquire an additional 8 h of time-lapse images. The migration speed increases for MCF-10A (Fig. 5A) and MDCK cells (Fig. 5C) after disruption of cell–cell adhesions by the EGTA treatment, whereas the migration speed of MDA-MB-231 cells (Fig. 5B) that contain lower amounts of cadherin at cell–cell junctions (Fig. 4E) is not affected by EGTA treatment. The effect of EGTA treatment on cadherin disruption is confirmed by immunostaining experiments (Fig. 5D). MCF-10A and MDCK tissues treated with EGTA are characterized by a very low amount of E-cadherins between cells (Fig. 5E), the amount of E-cadherins in EGTA-treated MDA-MB-231 tissues remain statistically unaffected. To strengthen our results, we add PEG at 2.5% (v/v) to EGTA-treated tissues. We observe that PEG treatment does not affect the migration speed of all three cell types (Fig. 5A–C), confirming that cadherin-based junctions are a key component of the migratory behavior of epithelial cell assemblies under moderate osmotic stress.

## Discussion

Many studies have shown that osmotic pressure can impact cell volume and affect cell structure^14,35,36^. Osmotic stress can lead to a significant reduction of the cell volume due to water efflux; this results in a corresponding increase in molecular intracellular crowding^14,77^. However, most of the previous work focused on isolated cells and used high levels of osmotic stress, both of which are far from physiological conditions.

Here, we study how moderate osmotic stresses can affect the migration of breast cell lines by using four different cell lines: a non-tumorigenic epithelial breast line (MCF-10A) and a malignant breast line with lack of E-cadherin (MDA-MB-231), a malignant breast line with positive E-cadherin expression (MCF-7) and a strong adhesion model of epithelial cells (MDCK). By performing OMTC experiments, we find that the mechanical properties of non-tumorigenic and tumorigenic breast cells remain constant under moderate osmotic pressure. That means that cell stiffness could not be considered as an important player in the modulation of the migratory behavior under osmotic stress.

To demonstrate the role of cell–cell adhesions, we compare the effect of osmotic pressure on cell volumes between single cells and cell clusters. Single cells are less affected than cell clusters, suggesting that cell–cell interactions have a key influence on modulation of cell volume in response to osmotic stress. To validate this hypothesis, we perform experiments with EGTA to inhibit cell–cell adhesion in cell clusters. We find that the cell volume is less affected by osmotic stress in EGTA-treated clusters, regardless of the cell type. This suggests that cell–cell adhesions have a major role in the cell-volume regulation mechanism and hence the sensitivity of epithelial cells to osmotic stress.

Our results suggest that tension across E-cadherin would change in response to osmotic conditions. Indeed, an essential aspect of epithelial homeostasis is attributed to maintenance of cell–cell junctions. In addition, changes in cell–cell junction are one of the hallmarks of epithelial-to-mesenchymal (EMT) transition which is an important regulator of cell migration. It could be therefore very interesting to use FRET-force biosensors for E-cadherin to estimate the tension in cell–cell junctions in response to the modulation of the osmotic conditions.

To conclude, this study helps unravel the variability in osmoregulatory mechanisms among different cell lines and highlights the central role of cell–cell interactions. We envision that a better understanding of the osmoregulation of cancer cells in different in vitro conditions might help to better target potential pharmacological agents.

## Material and methods

### Stamp fabrication

Stamps for micropatterning were fabricated by soft lithography using polydimethylsiloxane (PDMS; Sylgard 184 Silicone Elastomer Kit; Dow Corning). Briefly, a thin layer of SU8-3010 (MicroChem) was spin coated onto the surface of a silicon wafer. After baking, a photomask was placed on top of the wafer for UV exposure. Propylene glycol methyl ether acetate (PGMEA) (Sigma-Aldrich, 537,543) was then used to remove the unexposed SU8 from the wafer. After baking, the wafer was placed in a petri-dish and served as a mold for downstream PDMS fabrication. PDMS was well mixed with curing agent at a 10:1 ratio, degassed, poured onto the wafer previously passivated for 30 min with fluorosilane (tridecafluoro-1,1,2,2-tetrahydrooctyl-1-trichlorosilane). Finally, the wafer was placed in a 65 °C oven for at least 1.5 h. The PDMS was then cut and carefully peeled off from the mold.

### Cell culture

We used MDCK a renal epithelial cell model, MCF-10A a normal human breast epithelial cell and MDA-MB-231 a human tumor breast cell line. MDCK obtained from ATCC (ATCC CCL-34) were cultured in Eagle’s Minimum Essential Medium with 10% fetal bovine serum and 1% of penicillin/streptomycin at 37 °C with 5% CO_2_. MCF-10A cells obtained from ATCC (ATCC CRL-10317) were cultured in MBEM with additives obtained from Lonza/clonetics Corporation as a kit (MEGM, Kit catalog No. CC-3150) and 100 ng/ml of cholera toxin, 1% of penicillin/streptomycin at 37 °C with 5% CO_2_. MDA-MB-231 (ATCC HTB-26) and MCF-7 (ATCC HTB-22) were obtained from ATCC and cultured in Dulbecco's Modified Eagle Medium (DMEM) with 10% fetal bovine serum and 1% of penicillin/streptomycin at 37 °C. Cells were allowed to spread on substrates for several hours (> 4 h), then were synchronized by starvation in serum-free medium overnight before volume imaging and stiffness measurement. Cells were synchronized to avoid cell size growth and volume changes due to natural variations during the cell cycle^35^.

### Cell labeling and immunofluorescence

Live cells were fluorescently labeled with CellTracker (Invitrogen) to label cytoplasm and SiR-DNA (Sprirochrome) for nuclei, respectively. Cells were imaged and sectioned at 0.15-μm intervals by using excitation from a 633- or 543-nm laser or a 488-nm line of an argon laser and a × 63, 1.2-NA water immersion objective or × 40 dry air or × 20 dry air on a laser scanning confocal microscope (TCS SP5; Leica). To label cell–cell adhesions (E-cadherin, Beta-catenin and N-cadherin), cells were previously fixed with paraformaldehyde 4% and permeabilized with Triton-X100 (0.5%) in PBS and stained with 1/200 anti-E-cadherin-FITC (Invitrogen).

### Cell volume

Stained cells were observed by using a × 63/1.2-NA water immersion lens and tissue by using a × 20 dry lens on a Leica TSC SP5. Optical cross-sections were recorded at 0.15-μm z-axis intervals to show intracellular, nuclear, and cortical fluorescence. By using theoretical point spread function, a stack of gray-level images (8 bits) were subjected to deconvolution before 3D visualization. The 3D visualization was carried out by using ImageJ software. The volume was calculated by counting voxel number, after thresholding the stack. Confocal measurements were previously compared with AFM data and the results from the two techniques agreed.

### Osmotic stress

Hyperosmotic stresses were applied by adding PEG 300 to isotonic culture medium. The actual osmotic pressure applied to cells was calculated by adding the osmotic pressure due to PEG to that of isotonic solution (330 mOsm/kg) and was further confirmed through a selective measurement by using a micro-osmometer (model 3300; Advanced Instruments, Inc.). Before doing experiment, cells were allowed to equilibrate in PEG solution for 10 min at 37 °C and 5% CO_2_. The cell size decreases within 30 s and maintains the small volume for hours.

### Confocal microscopy imaging

To observe cell–cell adhesions, cells were seeded onto cell culture membrane assemblies and cultured in a cell incubator (37 °C and 5% CO_2_) for 16 h. Cells were fixed with 4% formaldehyde and 0.1% Triton × 100 diluted in PBS, followed by PBS washes for 3 times to remove excessive reagents. After cell fixation, cells were double stained for actin and E-cadherin antibody. Fixed cells were blocked with 10% bovine serum albumin (Thermo Fisher Scientific Inc, MA, USA) in PBS for 1 h, followed by a two-step immunostaining process for cadherin. Briefly, cells were first incubated with mouse monoclonal anti-cadherin antibody (Sigma-Aldrich, MO, USA) diluted × 200 in PBS with a supplement of 10% BSA for 45 min at 37 °C. Samples were then washed 5 times for 5 min each with PBS and incubated with goat anti-mouse Alexa Fluor 488 secondary antibodies diluted × 200 in PBS with a supplement of 10% normal goat serum for 1 h in the dark. To stain actin, Phalloidin-iFluor 555 (Abcam, MA, USA) are diluted at 1:400 to incubate cells for 1 h. Stained cells are then washed 3 times for 5 min with PBS and imaged with the confocal microscope. Cell–cell adhesions are stained with E-cadherin antibody. The image is obtained using × 25 or × 40 water immersion objective on a TCS-SP5 confocal laser scanning microscope (Leica Microsystems Inc., IL, USA).

### Cell tracking

Time-lapse microscopy experiments were performed with the Matlab pluggin Celltracker^46^ and analyzed with Prism Graphpad. The cell nucleus was stained with SirDNA (Spirochrome) to allow time-lapse microscopy experiments in live conditions for at least 16 h. We used a Leica TSC SP5 microscope equipped with × 20 magnification lens.

### Stiffness measurements

The cell mechanical properties were probed by using OMTC, which is a high-throughput tool for measuring adherent cell mechanics with high temporal and spatial resolution. Measurements were performed at 37 °C. In brief, functionalized ferrimagnetic beads (4.5 µm) coated with PLL (4 kDa) were incubated with cells for 20 min in the incubator. For stiffness measurements, beads were first magnetized horizontally by a large magnetic field. A much weaker magnetic field (oscillating at 0.75 Hz) was then applied vertically, which applied a sinusoidal torque to the beads. Motion of the beads was optically recorded. The ratio between the torque and bead motion thus defined an apparent cell stiffness (Pa/nm). A series of geometric factors, based on finite element models that take into account the cell thickness and bead-cell coupling, can be used to convert the apparent stiffness into shear modulus of the cell.

### E-cadherin contrast calculation

On the E-cadherin immunofluorescence images, an average intensity was measured at the cell−cell junction (i_jct_) and in the cytoplasm (i_cyt_) using ImageJ. These values where averaged over 20–25 cells (I_jct_ = ⟨i_jct_⟩ and I_cyt_ = ⟨i_cyt_⟩). We then defined J, the junction to cytoplasm ratio of E-cadherin intensity : J = I_jct_/I_cyt_.

### Cell viability

The four different cell lines are either incubated in medium with PEG 1, 1.5 or 2.5% or just medium as control to check the cell viability. Then, cells are trypsinised, centrifuged and resuspended in new media. 0.1 ml of solution of blue trypan 0.4% (Thermofisher) is added to 0.1 ml of cell solution. Then, blue cells are counted with a hemacytometer. % viable cells = [1.00 – (Number of blue cells ÷ Number of total cells)] × 100.

## Supplementary Information

Supplementary Information.

Supplementary Video 1.

Supplementary Video 2.

Supplementary Video 3.

Supplementary Video 4.

Supplementary Video 5.

Supplementary Video 6.

Supplementary Video 7.

Supplementary Video 8.
