# Supplementary material for: Tumorigenic mesenchymal clusters are less sensitive to moderate osmotic stresses due to low amounts of junctional E-cadherin
Source: Sci Rep. 2021 Aug 11;11:16279. doi: 10.1038/s41598-021-95740-x (PMC8358034; doi:10.1038/s41598-021-95740-x)
Supplement: Supplementary file 1 — Supplementary Information. [file 41598_2021_95740_MOESM1_ESM.docx]

**Tumorigenic mesenchymal clusters are less sensitive to moderate osmotic stresses due to low amounts of junctional E-cadherin**

**Danahe Mohammed^1,2^, Park Young Chan^3^, Jeffrey J. Fredberg^3^ and David A. Weitz^1,2*^**

^1^ John A. Paulson School of Engineering and Applied Sciences, Harvard University, Cambridge, Massachusetts, USA

^2^ Department of Physics, Harvard University, Cambridge, Massachusetts, USA

^3^ Harvard T.H. Chan School of Public Health, Boston, Massachusetts 02115, USA

*** Correspondence:**David A. Weitz

[weitz@seas.harvard.edu](mailto:weitz@seas.harvard.edu)


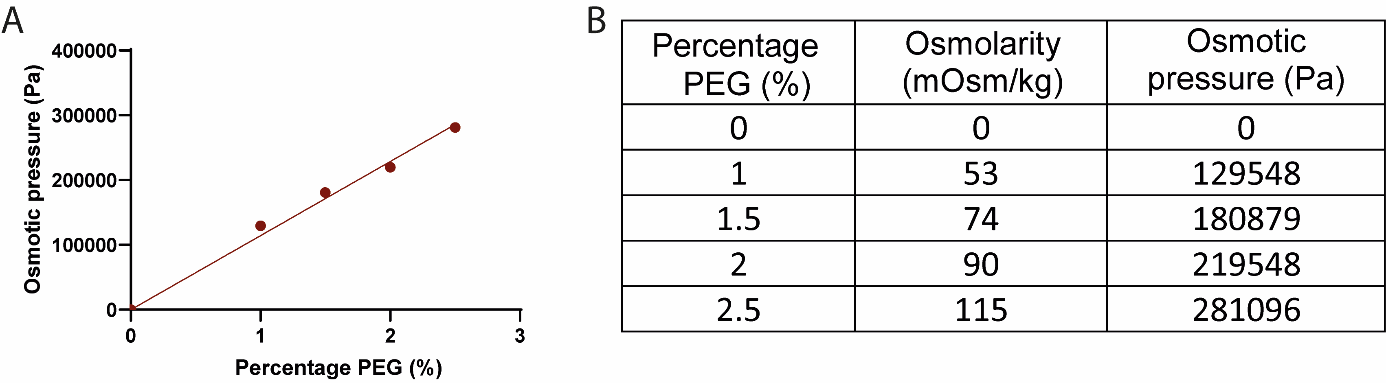


**Figure S1**. (A) Calibration curve of osmotic pressure measured in function of PEG concentration. (B) Table with values of osmolarity measured with an osmometer and the corresponding osmotic pressure.


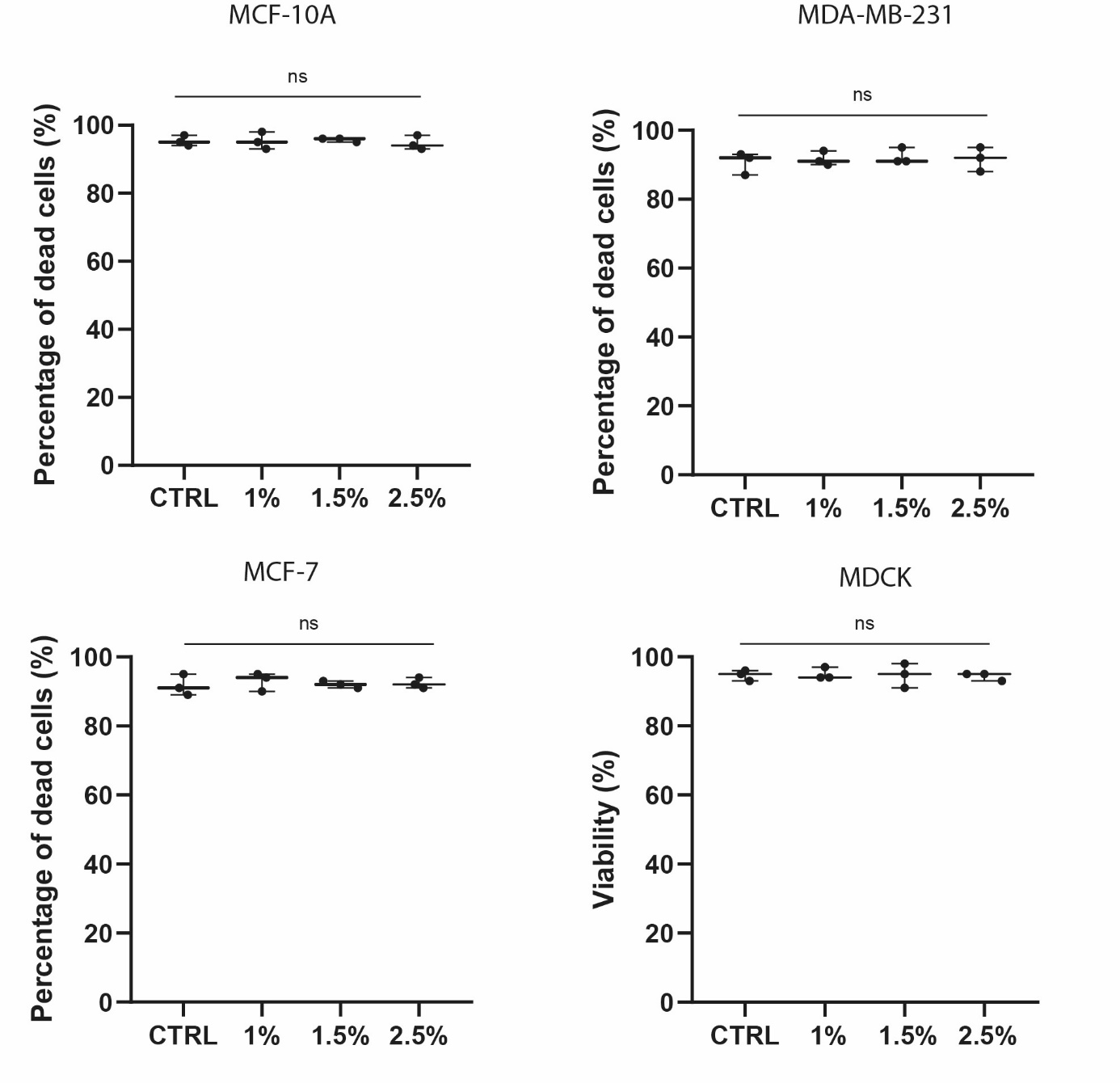


**Figure S2**. Cell viability under PEG treatments at 1, 1.5 and 2.5%. The control (CTRL) condition corresponds to the normal medium. n=3 independent experiments for each conditions and ns is non significant.


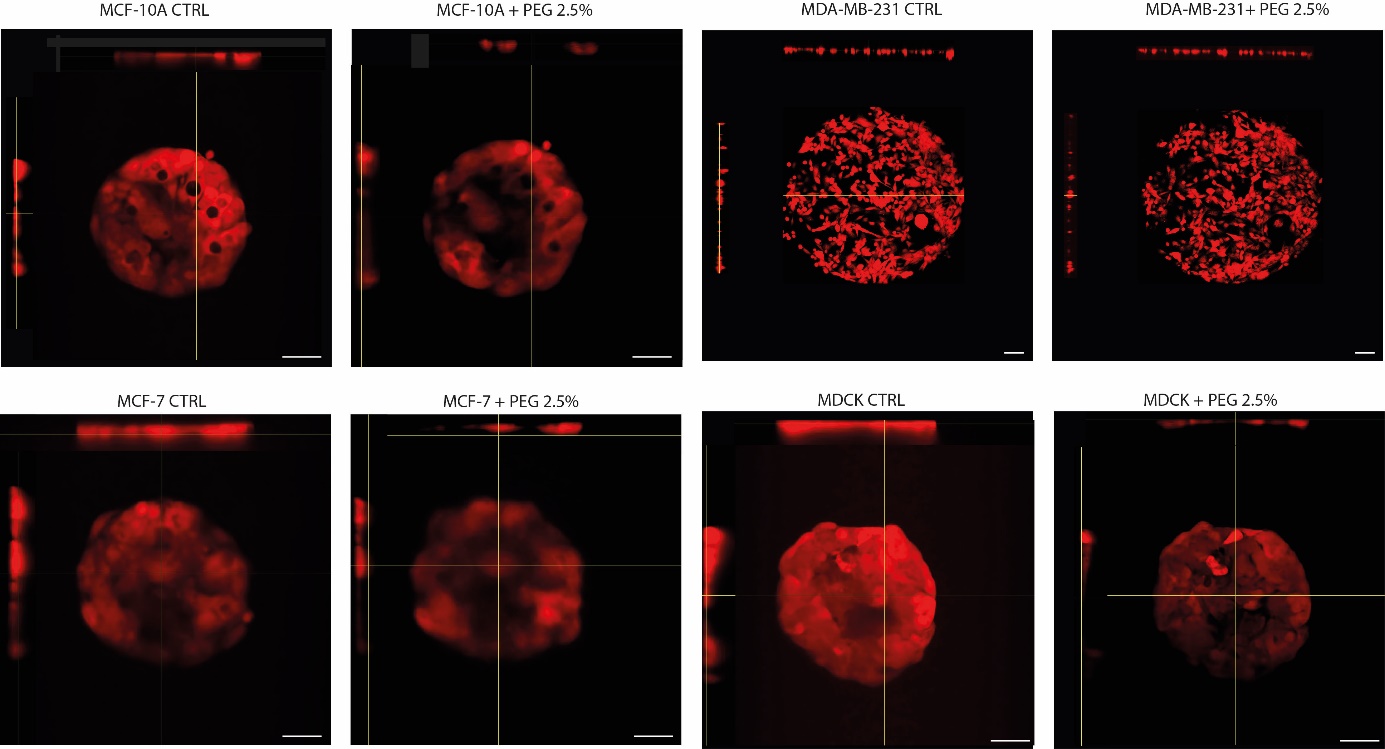


**Figure S3**. Confocal images with orthogonal views of tissues for control (CTRL) and PEG (2.5%) conditions. The cytoplasm is staining in live conditions with cell tracker (red).

**Movie S1.** Time-lapse sequence of fluorescent nucleus of MDCK clusters on glass surface. The trajectory of each cells is recorded. Total duration time: 8 hours.

**Movie S2.** Time-lapse sequence of fluorescent nucleus of MDCK clusters on glass surface with an addition of PEG 2.5% in the culture media. The trajectory of each cells is recorded. Total duration time: 8 hours.

**Movie S3.** Time-lapse sequence of fluorescent nucleus of MCF-10A clusters on glass surface. The trajectory of each cells is recorded. Total duration time: 8 hours.

**Movie S4.** Time-lapse sequence of fluorescent nucleus of MCF-10A clusters on glass surface with an addition of PEG 2.5% in the culture media. The trajectory of each cells is recorded. Total duration time: 8 hours.

**Movie S5.** Time-lapse sequence of fluorescent nucleus of MDA-MB-231 clusters on glass surface. The trajectory of each cells is recorded. Total duration time: 8 hours.

**Movie S6** Time-lapse sequence of fluorescent nucleus of MDA-MB-231 clusters on glass surface with an addition of PEG 2.5% in the culture media. The trajectory of each cells is recorded. Total duration time: 8 hours.

**Movie S7.** Time-lapse sequence of fluorescent nucleus of MCF-10A clusters on glass surface treated by EGTA. The trajectory of each cells is recorded. Total duration time: 8 hours.

**Movie S8.** Time-lapse sequence of fluorescent nucleus of MCF-10A clusters on glass surface treated by EGTA with an addition of PEG 2.5% in the culture media. The trajectory of each cells is recorded. Total duration time: 8 hours.
